# Exploratory investigation of changes in blood transcriptome following mepolizumab treatment for asthma

**DOI:** 10.3389/fimmu.2026.1839001

**Published:** 2026-06-15

**Authors:** Mitchell Pitlick, Mrunal K. Dehankar, Chantal E. McCabe, Sergio E. Chiarella, Kathleen Bartemes, Kay A. Bachman, Chung-Il Wi, Young J. Juhn, Thanai Pongdee

**Affiliations:** 1 Division of Allergic Diseases, Mayo Clinic, Rochester, MN, United States; 2 Allergy, Asthma, and Immunology Associates, Omaha, NE, United States; 3 Division of Computational Biology, Department of Quantitative Health Sciences, Mayo Clinic, Rochester, MN, United States; 4 Department of Otolaryngology - Head and Neck Surgery, Mayo Clinic, Rochester, MN, United States; 5 Precision Population Science Lab, Mayo Clinic, Rochester, MN, United States; 6 Department of Pediatric and Adolescent Medicine, Mayo Clinic, Rochester, MN, United States; 7 Office of Mayo Clinic Health System Research, Mayo Clinic Health System, Rochester, MN, United States

**Keywords:** asthma, eosinophil, mepolizumab, response, RNA, sequencing, transcriptome

## Abstract

**Objective:**

To evaluate the effects of mepolizumab therapy on the blood transcriptome in patients with severe eosinophilic asthma (SEA) and to determine whether changes in gene expression predict responsiveness to mepolizumab treatment.

**Methods:**

Fourteen consecutive patients diagnosed with SEA were prospectively enrolled. Each patient had sequential blood samples obtained pre-initiation and at 4, 8, and 12 weeks post-initiation of mepolizumab. Peripheral blood mononuclear cells (PBMCs) were isolated from blood samples for bulk RNA sequencing. Paired differential expression (DE) gene analysis was performed comparing baseline gene expression versus all timepoints. Changes in gene expression were also compared between responders versus nonresponders to mepolizumab treatment. Finally, gene set enrichment analysis was evaluated across timepoints and its interaction with the response to mepolizumab treatment.

**Results:**

Principal component analysis of all genes demonstrated a homogeneous distribution, which did not separate into distinct groups based on either timepoint or therapeutic response. At week 12, 149 genes had significant DE (FDR < 0.05) compared to baseline. When performing all-way timepoint comparisons, 74 genes (63 protein-coding) were found to be downregulated at weeks 4, 8 and 12 compared to baseline, but no differences were observed between post baseline timepoints. Twenty-eight genes (16 protein-coding) did change with progression of time and were associated with response to therapy, and 56 protein-coding genes distinguished responders and nonresponders. At week 12, nonresponders had a high enrichment of genes involved with signaling pathways, including cytokines, growth factors, cell differentiation markers, and protein kinases.

**Conclusion:**

In this exploratory study, mepolizumab use was associated with limited global transcriptomic separation with some preliminary differential-expression and enrichment findings. No definitive mechanistic or predictive response signatures were identified due to the study limitations. Future studies, including larger cohorts followed for longer durations with interrogation of specific immune subsets, may offer additional insight into the pathophysiology of severe eosinophilic asthma and into predicting response to mepolizumab treatment.

## Introduction

Asthma is a common, noncommunicable, chronic inflammatory lung disease affecting children and adults that is characterized by airway inflammation, hyperresponsiveness, and reversible airflow obstruction (1). For the past twenty years, asthma morbidity and mortality have been a critical problem of immense magnitude, with one in 20 adults and one in 10 children having asthma globally (2). In the United States alone, approximately 25 million individuals (7.7% of the population) currently have asthma, with almost 40% of these individuals experiencing an asthma exacerbation in the past year (3). Individuals with severe asthma represent about 10% of those with asthma, have significantly high healthcare utilization, and have increased risks of exacerbations, hospitalizations, and death (4).

Severe eosinophilic asthma (SEA) represents a subset of patients with severe asthma characterized by eosinophilic airway inflammation driven by the abnormal production of type 2 cytokines by T-helper 2 and innate lymphoid cells (5, 6). These cytokines include IL-4, IL-5, IL-13, and granulocyte macrophage colony-stimulating factor (5). Although SEA is clinically well recognized, much remains to be precisely characterized in the pathophysiologic mechanisms of the disease process (7). Recent advances have led to the development of several biologic treatments for SEA that target specific cytokine pathways, with anti-interleukin-5 (IL-5) therapy prominent among them given the role of IL-5 in eosinophil development, growth, and survival (1, 4, 8).

Much of the clinical benefit from anti-IL-5 therapy has been attributed to its direct effects on eosinophil depletion (1). However, other immunomodulatory effects stemming from anti-IL-5 therapy have been described that demonstrate a broader influence on the immune system beyond eosinophil depletion alone (9–13). These effects on the immune system are expected, as eosinophils play important roles in propagating diverse inflammatory pathways and modulating innate and adaptive immune processes (8). Investigation of changes in gene transcription resulting from anti-IL-5 therapy has been largely unexplored and is essential to gain further understanding of the immunologic pathophysiology of SEA. This may subsequently translate into improved diagnostic, prognostic, and therapeutic modalities that will better outcomes for patients with SEA.

Our study addresses the effects of anti-IL-5 therapy on the blood transcriptome by prospectively following patients with SEA who received mepolizumab over a 12-week period. Blood samples were collected prior to mepolizumab initiation as well as at multiple time points during the 12 weeks after the first dose of mepolizumab. These blood samples were then processed to isolate PBMCs for bulk RNA sequencing. Not only do we characterize differential gene expression in these patients, but we also evaluate whether changes in gene expression predict responsiveness to mepolizumab treatment.

## Materials and methods

### Patient cohort

The current study was approved by the Mayo Clinic institutional review board. Twenty consecutive patients who were diagnosed with SEA by a health care provider were consented and prospectively enrolled. In addition to a diagnosis of SEA, patients were also required to have a documented absolute blood eosinophil count ≥ 300/mm^3^ prior to enrollment. Enrolled patients had a prescription for mepolizumab provided during the course of routine clinical care, and enrollment occurred prior to the initiation of mepolizumab. Exclusion criteria included the following: 1) age <18 years old, 2) pregnancy, 3) use of any biologic or immunomodulatory therapy during or within 3 months of mepolizumab initiation, 4) any current or prior use of rituximab, 5) history of upper/lower respiratory tract infection or asthma exacerbation within the previous four weeks of the first baseline visit, 6) any prior history of malignancy, autoimmune disease, or immune deficiency, and 7) any other significant medical issue as determined by the principal investigator.

Each patient underwent sequential whole blood samples at four time points. The first time point occurred prior to receiving the initial dose of mepolizumab. Thereafter, blood samples were obtained at 4, 8, and 12 weeks post-initiation of mepolizumab (prior to each subsequent dose of mepolizumab). PBMCs were isolated for bulk RNA sequencing. After all blood samples had been collected, patients were classified as a “responder” or “nonresponder” after mepolizumab initiation. A “nonresponder” was defined as anyone who had an asthma exacerbation within 6 months of receiving their first dose of mepolizumab or a clinical history indicating poor control of asthma symptoms that resulted in switching to a different biologic therapy. A “responder” was anyone who did not meet the definition of a “nonresponder.”

### RNA sequencing

Total RNA concentration and quality were determined using Qubit fluorometry (ThermoFisher Scientific, Waltham, MA) and the Agilent Fragment Analyzer (Santa Clara, CA). The total RNA underwent globin depletion using GLOBINclear (ThermoFisher Scientific, Waltham, MA) and a clean-up with DNase I (Zymo Research, Irvine, CA) treatment. cDNA libraries were prepared using 100 ng of total RNA according to the manufacturer’s instructions for the Illumina Stranded mRNA Prep, Ligation (Illumina, San Diego, CA). The concentration and size distribution of the completed libraries were determined with the Agilent TapeStation and Qubit fluorometry. Libraries were multiplexed and sequenced in a single lane using the standard protocol for the Illumina NovaSeq 6000 and the NovaSeq XP 4-Lane kit for individual lane loading. The flow cell was sequenced as 100 X 2 paired-end reads using the NovaSeq S4 sequencing kit and NovaSeq Control Software v1.8.0. Base-calling was performed with Illumina’s RTA version 3.4.4.

### Data processing and analysis

The raw RNA sequencing paired-end reads were processed through the Mayo RNA-Seq bioinformatics pipeline, MAP-RSeq version 3.1.5 (14). MAP-RSeq uses STAR (15) to align reads to the reference human genome build hg38. Gene and exon expression quantification were performed using the Subread package (16) to obtain both raw and normalized (FPKM – Fragments Per Kilobase per Million mapped reads) reads. Finally, comprehensive analyses were run on the aligned reads to assess the quality of the sequenced libraries.

Using raw gene counts from MAP-RSeq, genes that were differentially expressed between the groups were assessed using the bioinformatics package edgeR 2.6.2 (17). Genes found to be different between the groups were reported along with their magnitude of change (log2 scale) and level of significance (False Discovery Rate, FDR < 5%). Canonical pathway analyses were performed using the Gene Set Enrichment Analysis (GSEA) and Ingenuity Pathway Analysis (IPA) (18). Pathways were reported along with their significance level (FDR < 25%). Next, we used limma-voom with duplicate correlation to model repeated-measures design, blocking on patient and applying empirical-Bayes moderation. Two analyses were fit: 1) timepoint-only model to estimate all pairwise timepoint contrasts, 2) response model with interaction to test timepoint effect, response effect, and the interaction of timepoint and response. To account for small sample size of non-responders, p value < 0.01 and |log2FC| >= 1 was used to determine genes with significant interaction. Single-sample GSEA (ssGSEA) was performed, and enrichment scores were compared across timepoints while adjusting for response, between response while adjusting for timepoint and for the interaction between timepoint and response.

## Results

Fourteen patients were enrolled (64% females) with a median age at time of mepolizumab initiation of 59.5 years old (IQR 45-67.8, range 29-87) and a median peak absolute eosinophil count prior to mepolizumab initiation of 640/mm^3^ (IQR 325-843, range 160-1450). Specific characteristics of the study cohort are summarized in **Table 1**. Analysis was performed on data from the enrolled 14 patients with a total of 54 samples. Two patients were missing one time point blood collection (54/56 samples were obtained). The study cohort included 2 patients classified as nonresponders and 8 patients classified as responders. Four patients had inadequate clinical data to be classified as a responder or nonresponder and were excluded from analyses testing the interaction effect of response to the drug across time points.

**Table 1:** Baseline clinical characteristics.

Subject #	Age at mepolizumab initiation (yrs)	Sex (M/F)	Peak AEC	FEV1	FeNO
1	65	F	620	101%	none
2	40	F	610	56%	23
3	78	M	1100	none	none
4	48	M	1060	60%	none
5	63	M	570	89%	16
6	87	M	1100	68% (on prednisone)	57
7	51	F	3600	90% (on prednisone)	32 (on prednisone)
8	62	F	540	55%	24
9	46	F	790	84%	238
10	42	F	1450	none	none
11	29	F	1080	88%	none
12	61	F	690	101%	29
13	58	M	1930	71%	none
14	76	F	610	57%	62

### Principal component analysis did not separate samples by timepoint or therapeutic response

**Figure 1:**
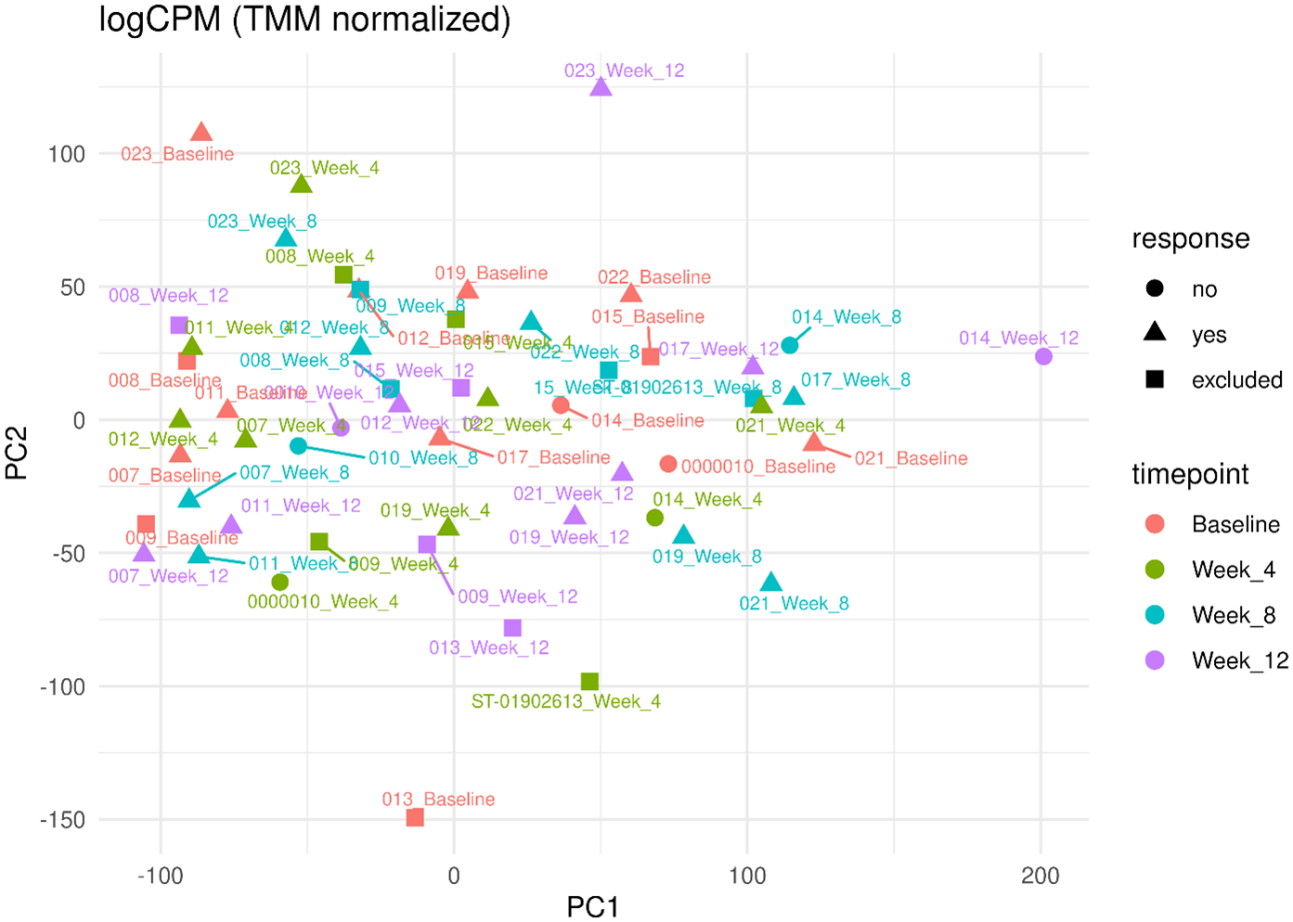
2D PCA. Principal component analysis shows samples do not cluster according to therapeutic response or timepoint of blood collection.

**Figure 2:**
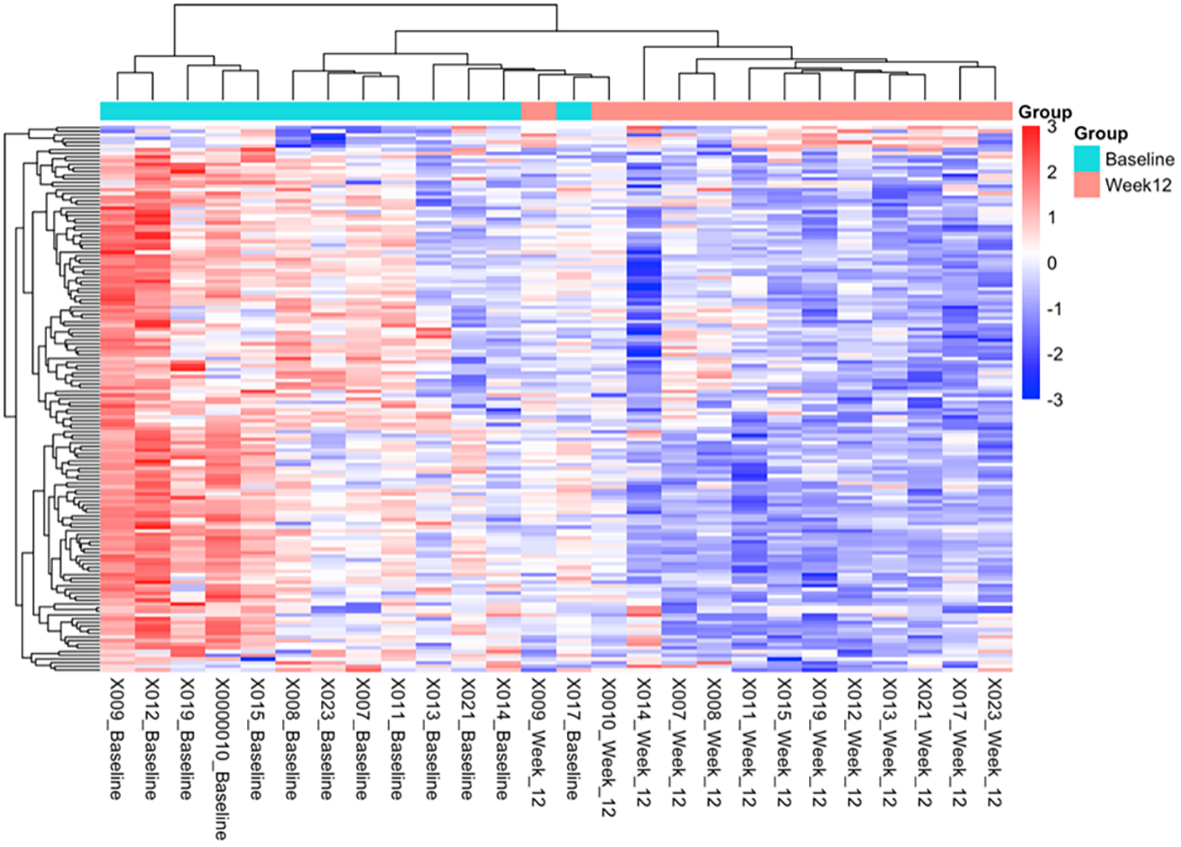
Paired DE results: baseline vs week 12. Blood samples collected at week 12 were compared to those collected prior to receiving initial dose of mepolizumab. 149 significant differentially expressed genes are observed (FDR < 0.05), with 6 genes upregulated at week 12 and 143 genes upregulated at baseline.

**Figure 3:**
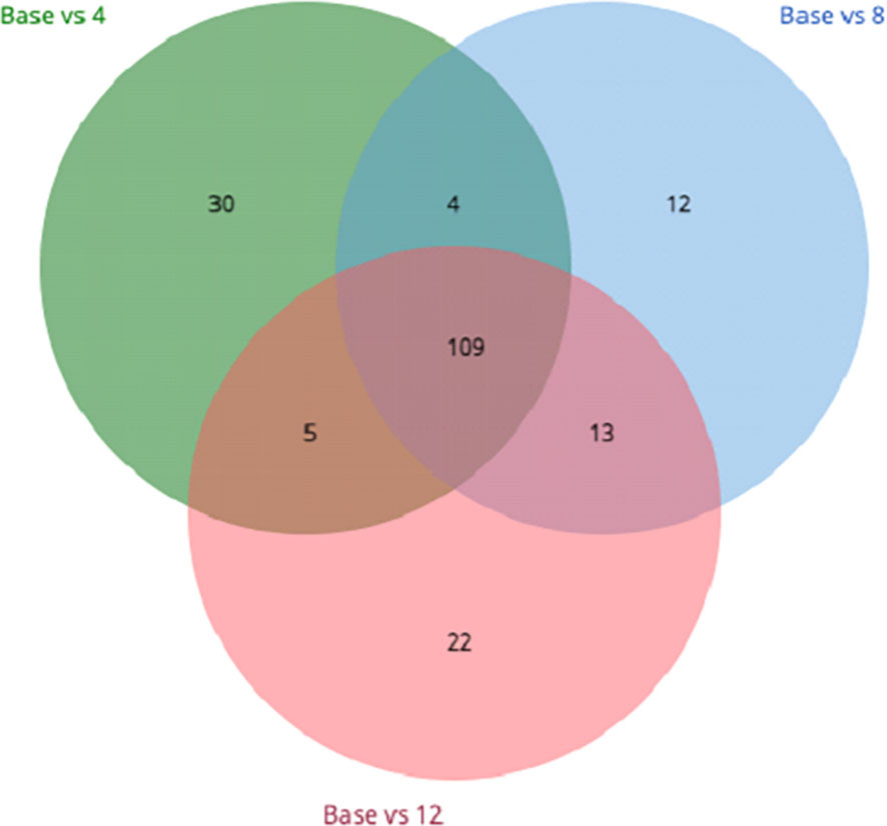
Venn diagram – DE genes. Venn diagram shows common and unique differentially expressed genes in paired group comparisons between baseline and 4, 8 and 12 post-initiation of mepolizumab. 109 genes were commonly differentially expressed at baseline compared to all other timepoints.

**Figure 4:**
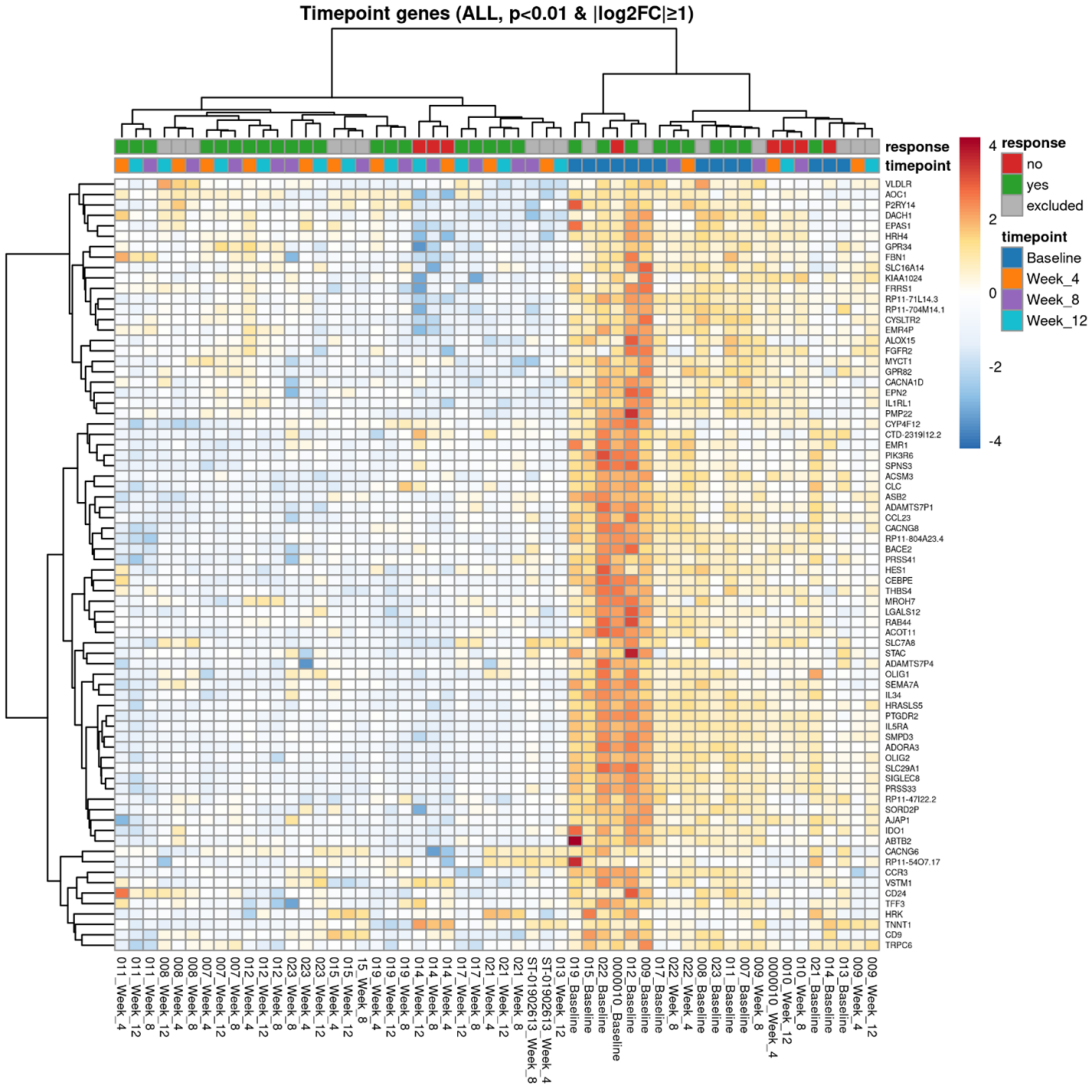
All-way timepoint gene comparisons (p<0.01 & │log2FC│≥1). Heatmap shows upregulation of 74 genes at baseline and no differences in expression of these genes at 4, 8 and 12 weeks post-initiation of mepolizumab.

**Figure 5:**
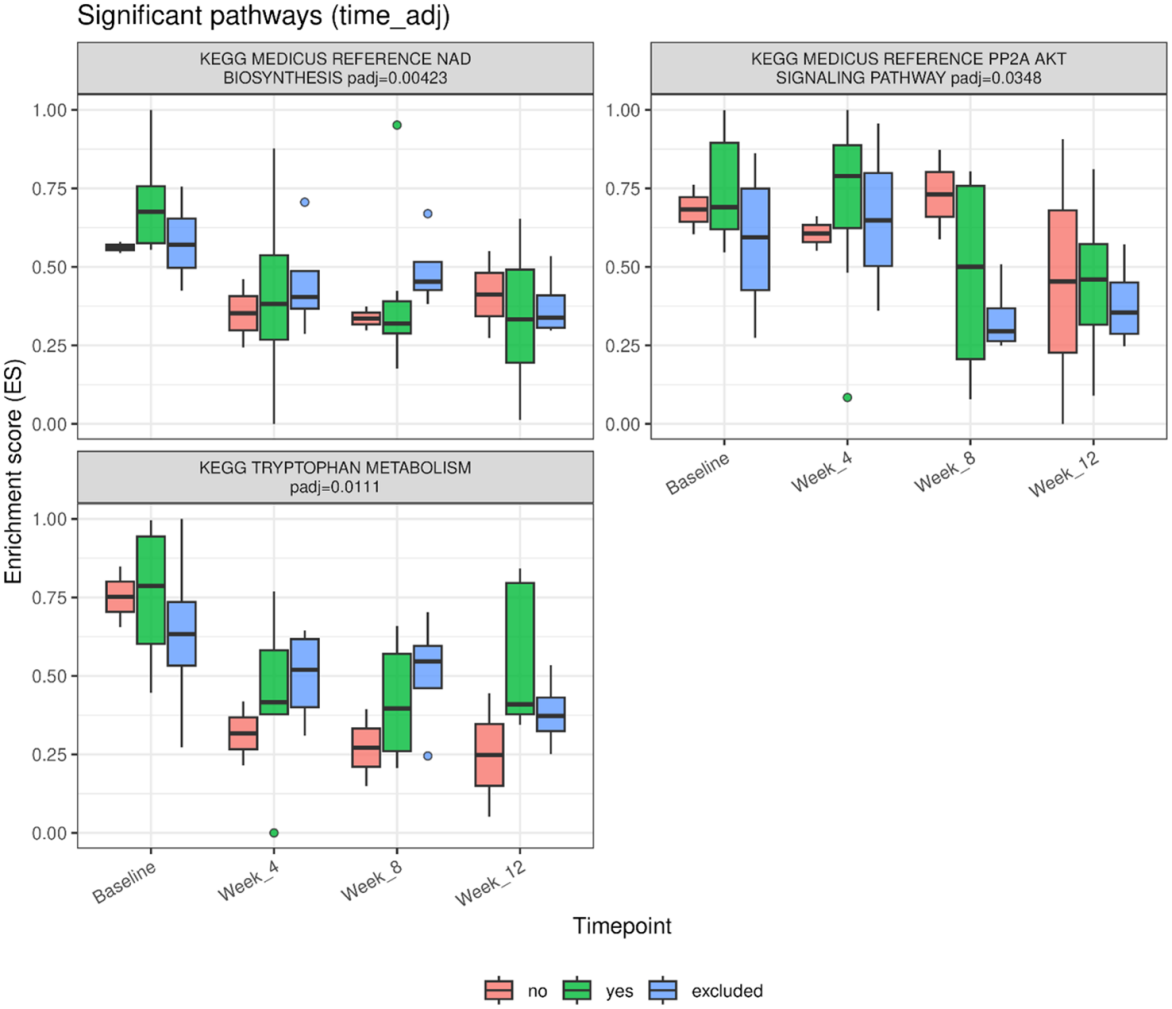
ssGSEA timepoint analysis, adjustment for response status. Single-sample gene set enrichment analysis shows pathways that change over time after adjusting for status for response to mepolizumab. Genes in NAD biosynthesis and tryptophan metabolism are enriched in baseline and depleted at subsequent time points; while PP2A AKT signaling pathway is enriched at early time points (baseline, weeks 4 and 8) and depleted at week 12.

Principal component analysis (PCA) of all genes demonstrated a homogeneous distribution which did not separate into distinct groups based on either timepoint or response (**Figure 1**). Paired differential expression (DE) gene analysis was performed comparing baseline gene expression versus timepoints at week 4, 8, and 12. At week 12, 149 genes (126 protein-coding) had significant DE (FDR < 0.05, **Figure 2**). Six genes had +Log2FC (higher expression at week 12) while 143 genes had -Log2FC (higher expression at baseline). The 126 DE protein-coding genes are listed in **Table 2**. Results were similar for the week 4 and week 8 timepoints compared to baseline. At week 8, 138 genes had significant DE (FDR < 0.05), and at week 4, 148 genes had significant DE (FDR < 0.05). The number of overlapping DE genes from the paired analyses are shown in **Figure 3** and are listed in **Table 3**. When performing all-way time point comparisons (**Figure 4**), 74 genes (63 protein-coding) were found to be downregulated at weeks 4, 8 and 12 compared to the baseline, but no differences were demonstrated between post baseline timepoints. Baseline samples formed a cluster of high to low expression, interspersed with some samples from other timepoints. Sixty-three protein-coding genes were found to be overexpressed at baseline compared to all time points. Of these 63 protein-coding genes, AOC1 and IDO1 genes belong to tryptophan metabolism, which was found to be significantly enriched at baseline (**Figure 5**). IDO1 also belongs to the NAD biosynthesis pathway found to be significantly enriched at baseline (**Figure 5**).

**Table 2:** Paired DE protein coding genes: baseline vs. week 12.

PLCH2	AJAP1	CNR2	PIK3R3	ACOT11	MROH7	FRRS1
CSF1	ADORA3	CD101	NTRK1	GPR137B	EPAS1	IL1RL1
INPP1	CDK15	FAM124B	SLC16A14	IL5RA	SRGAP3	CAMK1
STAC	CCR3	GRM2	CACNA1D	P2RY14	HES1	TEC
EREG	PAPSS1	C4orf33	CASP3	GAPT	THBS4	RHOBTB3
CPLX2	SYCP2L	GFOD1	RAB44	SLC29A1	CD24	MYB
MYCT1	RPS6KA2	HILPDA	AOC1	ASIC3	XK	GPR34
GPR82	ADAMDEC1	IDO1	VLDLR	PIP5K1B	GFI1B	ABO
PPP1R26	SWAP70	PLEKHA7	ABTB2	CAT	PTGDR2	ASRGL1
HRASLS5	LGALS12	P2RY2	TRPC6	DIXDC1	SCN4B	C10orf128
FGFR2	CD9	C3AR1	PKP2	METTL7A	FAM19A2	MSRB3
HRK	BRI3BP	CYSLTR2	DACH1	NDFIP2	TEX30	CEBPE
SLC7A8	ALDH6A1	TTC7B	ASB2	ZBTB42	SORD	FBN1
SEMA7A	KIAA1024	PRSS33	ACSM3	EEF2K	HSD3B7	LPCAT2
GPR114	SMPD3	IL34	SPNS3	ALOX15	MFSD6L	PIK3R6
PMP22	EPN2	CCL23	HRH4	PSTPIP2	SERPINB2	SLC24A3
EMR1	CYP4F12	KCTD15	CLC	SIGLEC8	CACNG8	CACNG6
VSTM1	TNNT1	MGAT3	OLIG2	OLIG1	BACE2	TFF3

**Table 3:** Overlapping DE genes from paired analyses.

AOC1	ACSM3	CD9	FGFR2	RPS6KA2	EPN2	IL5RA
CEBPE	SLC7A8	HSD3B7	ASB2	SMPD3	EEF2K	PRSS33
TNNT1	CLC	SIGLEC8	PIP5K1B	PMP22	SLC29A1	THBS4
HES1	IL1RL1	EPAS1	PIK3R3	ALDH6A1	MYCT1	CAT
ADORA3	CACNG6	IDO1	LGALS12	CAMK1	HRH4	HRK
TEC	TRPC6	CDK15	SEMA7A	SORD	CACNG8	STAC
GFOD1	VLDLR	DIXDC1	INPP1	CYSLTR2	FRRS1	IL34
CACNA1D	GPR114	TFF3	ALOX15	ACOT11	SLC16A14	RHOBTB3
GFI1B	ABTB2	FBN1	PLEKHA7	HRASLS5	KIAA1024	GPR82
EMR1	P2RY14	ABO	P2RY2	GAPT	SCN4B	ZBTB42
BACE2	SPNS3	PTGDR2	CCR3	OLIG1	MROH7	CSF1
MFSD6L	CYP4F12	CNR2	VSTM1	SRGAP3	FAM19A2	C10orf128
OLIG2	PRSS41	ADAMTS7P4	AC015971.2	GS1-421I3.2	RP11-618I10.1	RP11-47I22.2
RP11-704M14.1	RAB44	RP11-804A23.4	LINC00639	SORD2P	RP11-800A3.4	AC144831.1
CTD-2319I12.2	RP11-1018N14.5	EMR4P	RP11-430N14.4	RP11-215G15.5	ADAMTS7P1	CCL23
PIK3R6	DACH1	RP11-71L14.3				

### Limited global transcriptomic separation between responders and nonresponders

**Figure 6:**
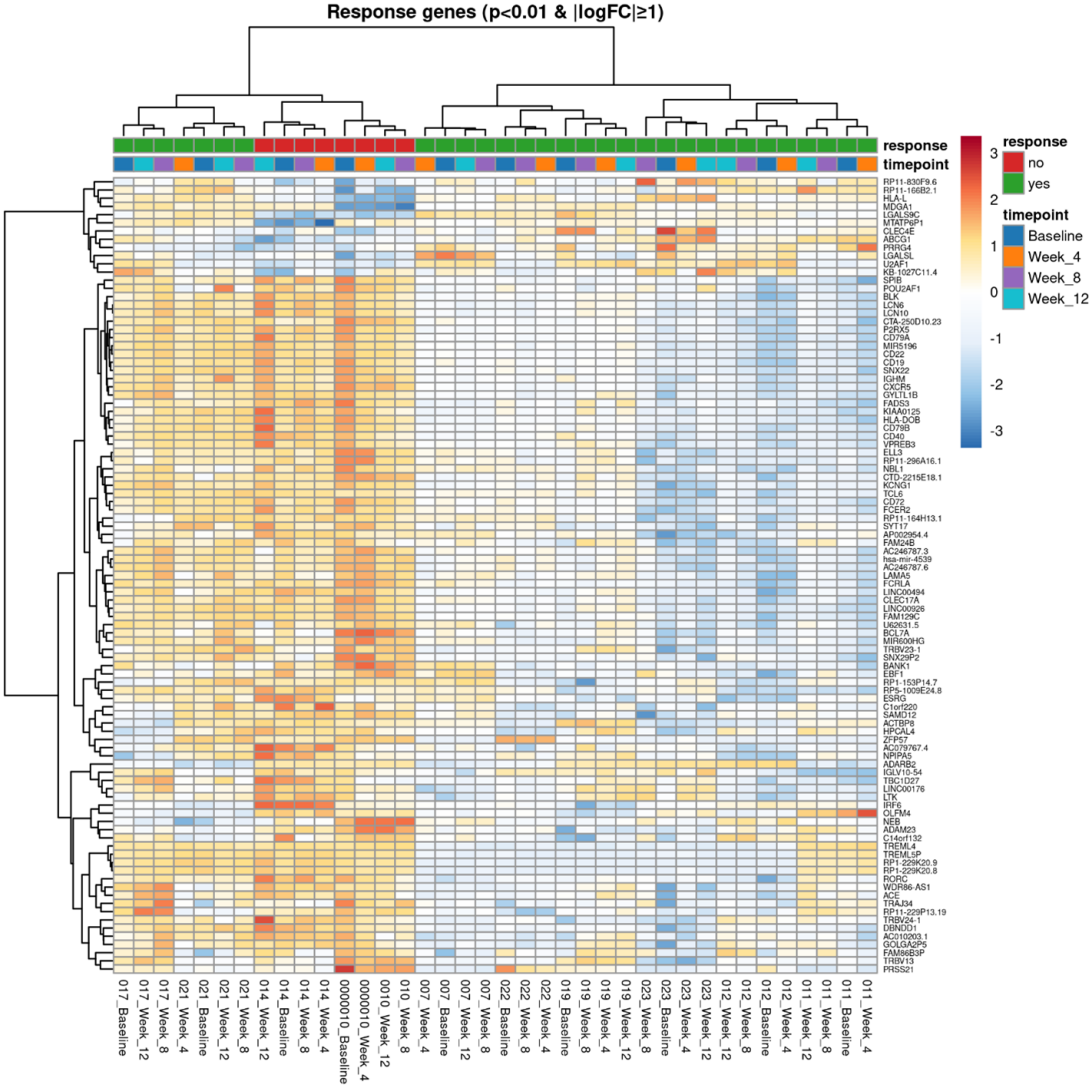
Genes distinguishing responders and nonresponders. Heatmap shows 97 significant differentially expressed genes between responders and nonresponders compared within each time point.

**Figure 7:**
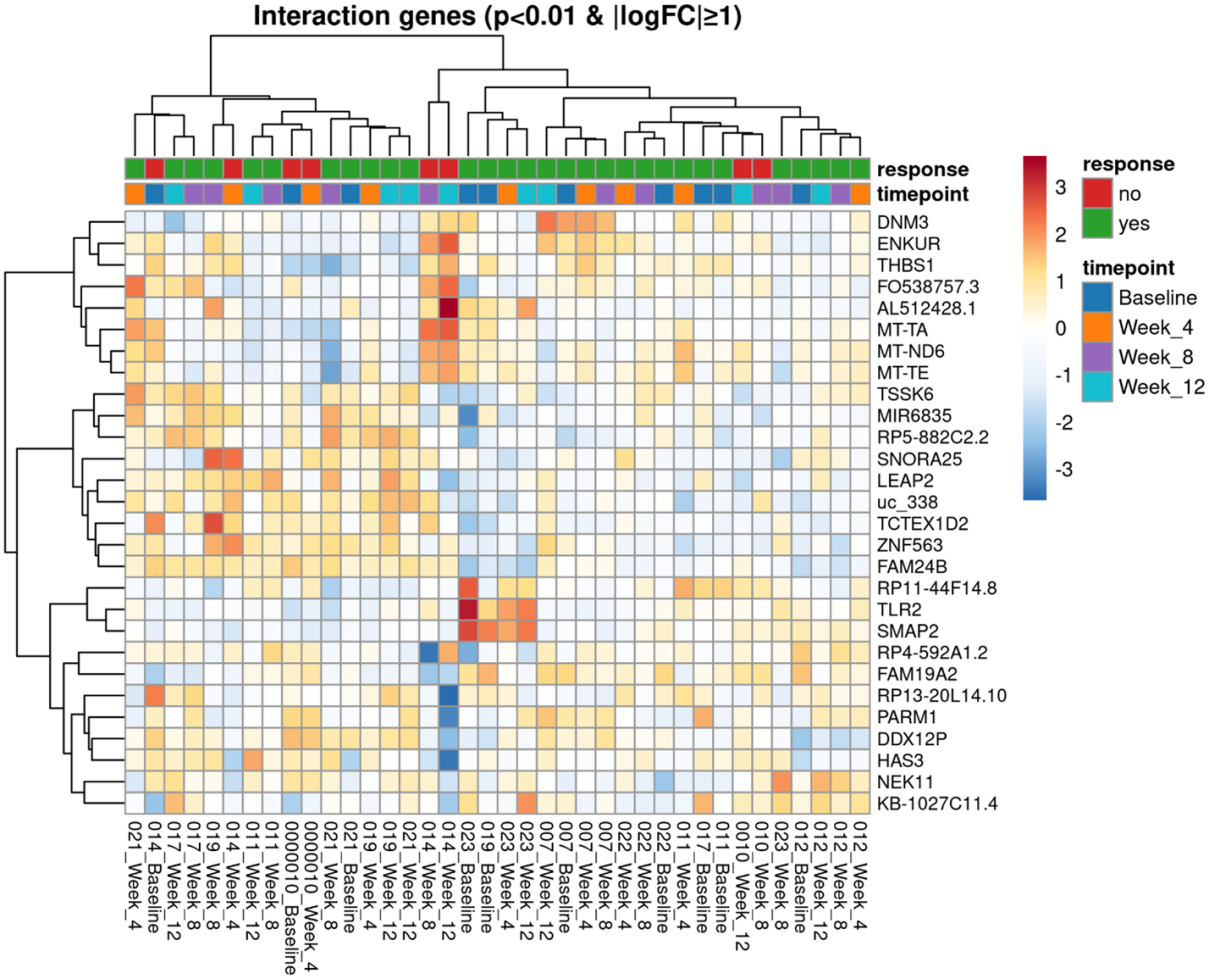
Timepoint x vs baseline changes in gene expression (p<0.01 & │log2FC│≥1). Heatmap shows change in gene expression in responders versus non-responders, compared at timepoint *x* versus baseline. Expression of 28 genes (16 protein coding) changes with progression of time and are associated with response to mepolizumab.

There were 97 genes (56 protein-coding) that distinguished responders from nonresponders. ADAM23, ADARB2, CXCR5, FCRLA, IRF6, OLFM4, SPIB, TREML4, and ZFP57 were upregulated in nonresponders at all time points, whereas MDGA1 and LGALS9C were downregulated in nonresponders at all time points. Additionally, we observed 2 responding patients clustering with and matching majority gene expression signature of nonresponders when subset to DE genes (**Figure 6**). Changes in gene expression in responders vs nonresponders were then compared at timepoint x versus baseline (**Figure 7**). There was generally no interaction demonstrated between response and timepoint variables. Expression of only 28 genes (16 protein-coding) changed with progression of time and were associated with response. Different magnitudes of change were observed in responders versus nonresponders at different timepoints. At week 12 compared to baseline, the following protein coding genes were upregulated in responders compared to non-responders: TSSK6, LEAP2, NEK11, TCTEX1D2, HAS3, PARM1; and the following were downregulated: MT-ND6, ENKUR, DNM3, THBS1. In week 8 compared to baseline, the following protein coding genes were upregulated in responders: ZNF563, TCTEX1D2, FAM24B; and the following genes were downregulated: TLR2, SMAP2. Finally, in week 4 compared to baseline, MT-ND6 and FO538757.3 were upregulated in responders, while FAM19A2 was downregulated in responders.

### Gene enrichment analysis demonstrates few significant differences in responders and nonresponders

**Figure 8:**
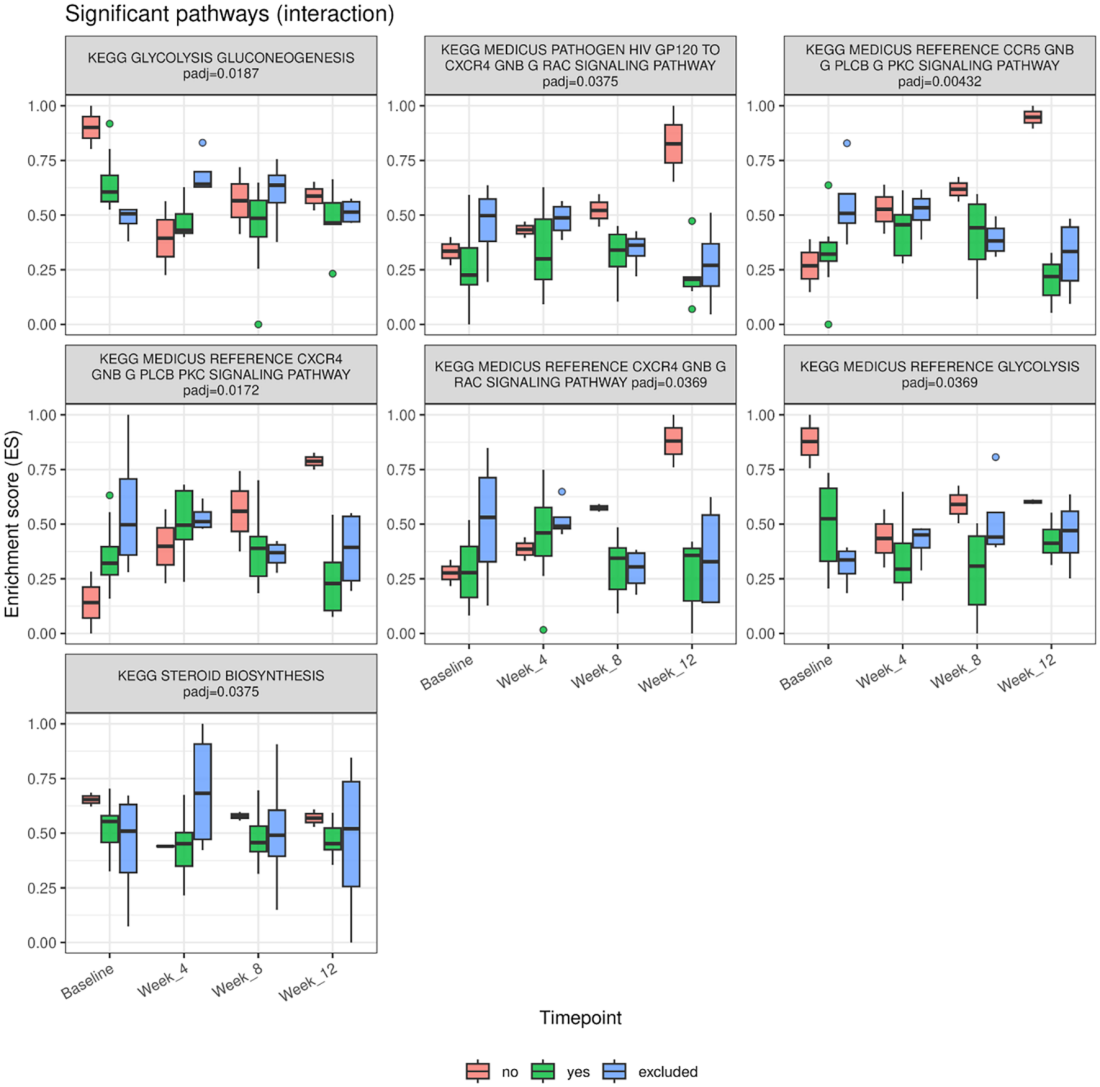
ssGSEA interaction analysis, response over time. Signaling and metabolic pathways that changed differently according to therapeutic response over time.

Single sample gene set enrichment analysis using the Kyoto Encyclopedia of Genes and Genomes (KEGG) did demonstrate a few significant differences in enrichment of KEGG pathways when evaluated across timepoints and its interaction with response. When evaluating interactions between response and timepoints, there were 7 KEGG pathways that changed differently according to response over time, representing signaling and metabolic pathways (**Figure 8**). At week 12, nonresponders had a high enrichment of genes in CCR5-GNB/G-PLCB/G-PKC, HIV gp120 to CXCR4-GNB/G-RAC, CXCR4-GNB/G-PLCB-PKC and CXCR4-GNB/G-RAC signaling pathways. In metabolic pathways, the nonresponders have high enrichment of glycolysis pathways at baseline. When evaluating KEGG pathways that changed over time, with adjustment for response status, 3 KEGG pathways changed over time: NAD biosynthesis, PP2A-AKT signaling, and tryptophan metabolism (see **Figure 5**). NAD biosynthesis and tryptophan metabolism genes have high enrichment at baseline in responders and non-responders with decreased enrichment at later time points. The PP2A AKT signaling pathway is seen to be enriched at early time points (baseline, weeks 4 and 8), and diminishes at week 12 in responders and non-responders.

## Discussion

This study is one of the few to investigate the effects of mepolizumab on gene expression in patients with severe eosinophilic asthma and whether changes in gene expression may predict responsiveness to treatment with mepolizumab. As our work was exploratory in nature, we demonstrated limited global transcriptomic separation with some preliminary differential-expression and enrichment findings of interest. While several genes did have differential expression at each time point post-baseline and several genes were over-expressed at baseline compared to all time points, there were no significant differences between post baseline timepoints. Twenty-eight genes (16 protein-coding) did change with the progression of time and were associated with response, and 56 protein-coding genes distinguished responders and nonresponders. We were unable to detect a significant difference in genes expressed when accounting for different time points, but there were a few differences in gene enrichment for protein-coding genes that were associated with response to mepolizumab. Gene enrichment analysis highlighted a few signaling and metabolic pathways associated with asthma inflammation that may warrant further investigation. Given the exploratory nature of our study and our study design, no definitive conclusions may be made regarding the observed gene differences or regarding which cell populations and mechanistic pathways are driving the observed transcriptomic changes.

In terms of potential immune mechanisms to further characterize, gene enrichment analysis demonstrated some significant differences in enrichment of KEGG pathways when evaluated across timepoints and its interaction with response. At week 12, nonresponders had a high enrichment of genes in the CCR5-GNB/G-PLCB/G-PKC, HIV gp120 to CXCR4-GNB/G-RAC, CXCR4-GNB/G-PLCB-PKC and CXCR4-GNB/G-RAC signaling pathways. In relation to asthma, each of these signaling cascades may adversely affect asthma outcomes, thereby correlating with a nonresponder phenotype. The CCR5-GNB/G-PLCB/G-PKC signaling pathway has been implicated to be involved with T1 in addition to T2 inflammation with associated resistance to corticosteroid treatment (19, 20). The HIV gp120 to CXCR4-GNB/G-RAC has been linked to mucus formation in human bronchial epithelial cells (21). Finally, the CXCR4-GNB/G-PLCB-PKC and CXCR4-GNB/G-RAC signaling pathways have demonstrated associations with immune-mediated airway inflammation and airway hyperresponsiveness in asthma (22–24). With our small sample size, short-term follow-up, and other study limitations, these response-related findings are exploratory and should not be interpreted as validated predictive markers.

In addition to the differences in signaling pathways, KEGG pathway gene enrichment analysis also demonstrated changes in metabolic pathways across timepoints. Notably, nonresponders had a high enrichment of glycolysis pathways at baseline. This finding correlates with a nonresponder phenotype, as recent studies have noted that enhanced glycolytic activity in Th2 cells, airway epithelial cells, and airway smooth muscle cells promote inflammation and remodeling in asthma (25, 26). When evaluating other metabolic pathways, 3 KEGG pathways changed over time including NAD biosynthesis, PP2A-AKT signaling, and tryptophan metabolism with enrichment at baseline and depletion at week 12 in both responders and nonresponders. Each of these pathways has relevance to asthma pathophysiology. In prior studies, NAD metabolism has demonstrated associations with type 2 inflammation and eosinophilic phenotypes (27, 28). PP2A-AKT signaling has been shown to be involved in eosinophil migration, activation, and survival as well as in airway hyperresponsiveness (29–31). Lastly, tryptophan metabolism has been associated with airway inflammation, hyperresponsiveness, and remodeling (32). In addition, tryptophan metabolism may also influence the balance between Th1 and Th2 immune responses (33). These findings regarding differences in metabolic pathways are exploratory in nature and lack validation as predictive biomarkers.

Prior studies have utilized metabolomics to identify potential biomarkers for distinct asthma phenotypes as well as to aid selection of biologic therapy (34–37). One prior study demonstrated specific metabolites associated with response to mepolizumab. Nopsopon et al. (38) performed untargeted metabolomic profiling on pre-treatment plasma samples from 31 patients with moderate-to-severe asthma who initiated mepolizumab and were followed for 12 months. Of 1150 measured metabolites, only 4 metabolites demonstrated statistical significance after FDR correction. Two tocopherol metabolites, δ-carboxyethyl hydroxychroman (CEHC) (FDR = 0.01) and δ-CEHC glucuronide (FDR = 0.0028), were associated with more frequent exacerbations while receiving mepolizumab (nonresponder). Two bilirubin degradation products (FDR of 0.005 and 0.04, respectively) were associated with fewer exacerbations when on mepolizumab (responder). In relation to our study, Nopsopon et al. (38) found that kynurenate, a tryptophan metabolite (uncorrected p=0.002), was associated with higher exacerbations on mepolizumab, but not after FDR correction, while metabolites related to NAD biosynthesis and PP2A-AKT signaling were not described in the results.

Another metabolomic study by Contreras et al. (39) monitored mepolizumab treatment using a targeted approach evaluating 35 metabolites associated with allergic inflammation. In this study, 36 patients with severe asthma were followed for 18 months after starting mepolizumab with serum samples collected at baseline, 6 months and 18 months after treatment initiation. After multivariate analysis, arachidonic acid, palmitoleic acid, oleic acid, propionylcarnitine, bilirubin, CCL11, and TNFSF10 significantly correlated with clinical improvement at 18 months. The targeted panel did not allow for comparison with the metabolic pathways identified in our study. As both Nopsopon et al. (38) and Contreras et al. (39) reported significant changes in measured biomarkers after 12 months of treatment, the short time frame for sampling in our study poses a potential limitation to detect differences in the transcriptome.

Few other studies have investigated whether transcriptomics may identify gene expression signatures that distinguish patients treated with mepolizumab in terms of immune system changes or responsiveness to treatment. Van Hulst et al. (40) performed RNA sequencing for eosinophils isolated from 10 patients with severe eosinophilic asthma treated with mepolizumab for ≥ 6 months and from 10 healthy volunteers. Gene expression profiles from sample clustering and PCA analyses failed to distinguish the patients treated with mepolizumab from healthy patients. Pairwise differential gene expression analyses also demonstrated no differentially expressed genes between the subject groups. Based on these results, gene expression profiles from residual blood eosinophils from patients with severe eosinophilic asthma were not distinctive from those of healthy patients. Van Hulst et al. (40) also isolated eosinophils from an additional 3 patients with severe eosinophilic asthma treated with mepolizumab and stimulated the eosinophils with IL-33 for 6 hours before processing for RNA sequencing. Principal component analyses indicated that observed changes in the eosinophil transcriptome were predominantly due to IL-33 stimulation and not due to mepolizumab. These findings suggest that mepolizumab has little impact on gene expression of residual steady-state eosinophils (40).

Park et al. (41) used single-cell RNA sequencing to investigate transcriptional changes in circulating immune cells from patients treated with biologics for their severe asthma. In this study, PBMCs were isolated from 8 patients with severe eosinophilic asthma before and after treatment with mepolizumab (n=2), reslizumab (n=2), or dupilumab (n=4). Transcriptional profiles did not significantly change after the first month of treatment. However, after 6 months of treatment, several changes were noted. The composition of classical monocytes (CM) changed with a reduction in IL1β+ CMs and an increase in S100A+ CMs. There was also significant suppression of the NF-kB pathway across multiple immune cells, including T, B, NK, and myeloid cells. These changes were noted regardless of the biologics used (41). Responsiveness to biologic therapy was not assessed in this study.

Rakkar et al. (42) investigated whether changes in the nasal transcriptome in those with asthma could predict response to mepolizumab at 1 year. In this study, 27 patients with asthma (n=17 responders, n=10 nonresponders) were enrolled, and responders were defined as patients who had a 50% reduction in exacerbation frequency and/or 50% reduction in oral corticosteroid use after 1 year of treatment with mepolizumab. Nasal brushes were taken at baseline before initiation of mepolizumab and after 3 months of treatment. At baseline, there were no significant differences in gene expression between responders and nonresponders. Differential gene expression analysis between baseline and after 3 months of mepolizumab treatment revealed 1,784 genes unique to responders and 893 genes unique to nonresponders, with 1,500 genes overlapping. In nonresponders, mepolizumab treatment was associated with changes in gene expression for active upstream regulators TSLP, IL-4, and IL-33. In addition, IL-1α, IL-1β, and IFN-α2, were inhibited in nonresponders. These results suggest that mepolizumab significantly affects the upper airway epithelium inflammatory profile, and a 12-week gene expression profile may predict response to therapy at 1 year (42).

Another related study analyzed gene expression from RNA sequencing data to characterize asthma exacerbations. Altman et al. (43) reported a secondary analysis of the MUPPITS-2 study, a double-blind, placebo-controlled, randomized clinical trial comparing mepolizumab treatment versus placebo among urban children with exacerbation-prone asthma (44), involving RNA sequencing performed on bulk RNA extracted from nasal lavage cell pellets. In this analysis, treatment with mepolizumab demonstrated differing levels of gene expression in T2 and non-T2 inflammatory pathways during illness events resulting in asthma exacerbations when compared to children receiving placebo. Findings from this study provide further understanding into potential mechanisms for reduced clinical responses to mepolizumab based on the airway transcriptome (43).

These prior studies all indicate that mepolizumab likely alters the transcriptome in a meaningful way that may offer insight into the pathophysiology of eosinophilic asthma, along with indications about responsiveness to treatment. Our exploratory, hypothesis-generating study offers potential additional mechanistic pathways to consider for future investigation, but our study has several limitations to note. First, the study cohort, being exploratory in nature, was small with only 14 total patients and only 10 could be included in the response analysis. Therefore, the response analysis is underpowered and not suitable for predictive inference. In addition, the 12-week sampling window is not fully aligned with the longer-term clinical response definition. The study design was based on real-world practice with mepolizumab prescribed as part of routine clinical care and patient follow-up was based on clinical practice. Accordingly, we had no control group for comparison. Consequently, there are several variables related to asthma status that were either not controlled or not measured in a systematic manner. These uncontrolled or unmeasured variables may be potentially confounding the differences between time points. Furthermore, the use of bulk RNA sequencing of PBMCs does not resolve the cellular source of the observed transcriptomic signal and may be obscuring changes in gene expression in individual cell types at different time points and in terms of responder status. In addition, PBMC profiling cannot distinguish whether the observed signal reflects transcriptional regulation within specific immune cell subsets versus shifts in circulating cell composition, as changes in eosinophil-related compartments may indirectly influence the blood transcriptome. Future studies will require larger, better-defined, and more controlled cohorts. Single-cell RNA sequencing may also be needed to provide higher resolution characterization of treatment-induced immune system and gene expression changes, and independent validation will be required before any clinical interpretation can be supported.

Overall, our study demonstrated that mepolizumab use was associated with only limited global transcriptomic separation with some exploratory differential-expression and enrichment findings. These exploratory findings included signaling and metabolic pathways associated with asthma inflammation and may warrant further investigation. Future studies that address the limitations of our study, including larger cohorts followed for longer durations with interrogation of specific immune subsets, may offer additional insight into the pathophysiology of severe eosinophilic asthma and into predicting response to mepolizumab treatment.

## Data Availability

The data presented in the study are deposited in the GEO repository, accession number GSE334345.
